# Potential global distribution area projections of the aphid *Lipaphis erysimi* and its predator *Eupeodes corollae* in the context of climate change

**DOI:** 10.3389/fpls.2022.1019693

**Published:** 2022-11-25

**Authors:** Yuyang Lian, Aqiang Wang, Sihua Peng, Jingjing Jia, Xiaofeng Yang, Jinlei Li, Shuyan Yang, Rongjiao Zheng, Shihao Zhou

**Affiliations:** ^1^ Sanya Nanfan Research Institute of Hainan University, Sanya, Hainan, China; ^2^ Key Laboratory of Germplasm Resources Biology of Tropical Special Ornamental Plants of Hainan Province, College of Forestry, Hainan University, Haikou, Hainan, China; ^3^ Institute of Plant Protection, Hainan Academy of Agricultural Sciences (Research Center of Quality Safety and Standards for Agricultural Products of Hainan Academy of Agricultural Sciences), Haikou, Hainan, China

**Keywords:** *Lipaphis erysimi*, *Eupeodes corollae* Fabricius, Suitable area, Climate factor, MaxEnt

## Abstract

Climate change affects the population distribution of pests and their natural enemies, and predicting these effects is necessary for pest monitoring and green control. *Lipaphis erysimi* is an important vegetable pest, and its natural enemy, the *Eupeodes corollae* Fabricius has a strong predatory effect on the *L. erysimi*. To assess the spread trends of *L. erysimi* and its natural enemy, the hoverfly, *E. corollae* under current (1970-2000) and future climates (2041-2060), based on the MaxEnt model, this paper uses data on the geographical distribution of the historical occurrence of *L. erysimi* and *E. corollae* to speculate on their potential distribution areas worldwide and analyze the key environmental factors affecting the survival and spread of both. The results showed that the Representative Concentration Pathway (RCP) 2.6 and RCP4.5 climatic conditions are favorable for the spread of *L. erysimi*, the RCP8.5 climatic conditions are unfavorable for the spread of *L. erysimi*, and all three future climatic conditions are unfavorable for the spread of *E. corollae*. The highest fitness of *L. erysimi* was found at the annual average temperature of 18 °C and the annual average precipitation of 900 mm, while the highest fitness of *E. corollae* was found at the annual average temperature of 10 °C and the lowest temperature in the coldest month of 0 °C. This study can provide a reference basis for monitoring and early warning and biological control of *L. erysimi*.

## Introduction

*Lipaphis erysimi* (Kaltenbach) (Homoptera: Aphididae) mainly harms cruciferous crops such as radish, rape, and cabbage, and is particularly harmful to Brassica crops such as mustard and rape (Rana, 2005). It was first discovered in the United States by Davis (1914) and is now widely distributed worldwide. *L. erysimi* are reproduce quickly and usually cluster in groups on the young stems and abaxial surface of leaves to feed on sap, seriously affecting the growth and development of vegetables, the secreted honeydew can cause sooty blotch, prevent the leaves from photosynthesis. In addition, *L. erysimi* can act as a mediating insect to transmit a variety of plant viruses, resulting in slow growth, yellowing and wilting of leaves, and even death of the entire plant (Koramutla et al., 2016). Rape is one of the major oil crops in the world, and the *L. erysimi*’s damage has caused huge losses to the economy (Chattopadhyay et al., 2005). The larvae of *Eupeodes corollae* Fabricius (Diptera: Syrphidae) are an important predatory natural enemy of *L. erysimi* (He et al., 1990), they feed heavily and are the dominant species in many locations, making them ideal for control of the *L. erysimi* (Pekas et al., 2020).

Global warming has become one of the main features of today’s climate change, and the Earth’s climate is expected to become hotter and more humid in the coming century than it is today (Barreca, 2012). Insects are sensitive to environmental changes because of their small size, thin body walls, rapid heat exchange with the environment, and poor ability to self-regulate body temperature (Yamamura et al., 2006). Temperature and humidity are important factors affecting the growth, development, survival, and reproduction of individual insects and populations, and adaptation to temperature and humidity is a key condition necessary for insects to carry out their life activities (Khaliq et al., 2014). The maximum and minimum temperatures of 27.37°C and 14.62°C, maximum and minimum relative humidity of 95.28% and 62.28%, respectively, were detected at the peak of *L. erysimi* occurrence, and the population density of *L. erysimi* decreased with the increase of humidity (Reza et al., 2004). The developmental starting temperatures for eggs, the first instar larvae, the second instar larvae, the third instar larvae, and pupae of the *E. corollae* are 9.69°C, 12.39°C, 6.97°C, 2.03°C, and 2.35°C, respectively, and temperatures above 30°C result in mass mortality of larvae and failure of pupae to fledge (Dong et al., 2004). The fledge rate can reach 90% when the soil humidity is 75%-90%, and lower than 60% will remarkably reduce the fledge rate (Li et al., 1996). Temperature and humidity are crucial to the population dynamics of *L. erysimi* and *E. corolla*. Therefore, we selected *E. corolla* as predatory natural enemies of *L. erysimi* to study the changes of their potential distribution area in different climatic conditions.

Ecological niche models are based on ecological niche theory, which analyzes known species distribution data and their associated environmental variables to predict the potential distribution of species (Sillero, 2011; Yackulic et al., 2013). The maximum entropy (MaxEnt) ecological niche model is a machine learning modeling approach based on the maximum entropy principle that uses only data related to environmental variables and habitat suitability to simulate species ecological niche, and estimates the distribution of potential fitness zones of specie’s by determining the maximum entropy distribution constrained by environmental variables (Elith et al., 2011; Warren and Seifert, 2011), the output of which is a distribution map reflecting different fitness levels of species (Zhao et al., 2022b). MaxEnt’s principle-based algorithm can obtain the most uniform potential distribution of species and can provide highly accurate predictions compared to other prediction models, even when species distribution data are small or incomplete. In addition, the output of the MaxEnt model combined with GIS can describe the weight of each environmental factor affecting the expected distribution of species, so that the dominant environmental parameters affecting species distribution can be obtained (Phillips et al., 2006; Pearson et al., 2007; Liu et al., 2018). Dong et al. (2022) used the MaxEnt model to predict the change of potential suitable areas of *Bactrocera dorsalis* (Hendel). The results showed that the suitable areas for *B. dorsalis* will increase, and the range will likely expand northward from existing locations in the future. Using MaxEnt to predict the distribution of *Bactrocera correcta*, it was concluded that its potential suitable areas include India and neighboring countries in Asia, pacific islands, and North Australia, Central and South America, central Africa. Water vapor pressure and solar radiation were the most influential variables for *B. correcta*, the rising temperature could lead increasing of suitable area slightly (Zhang et al., 2022b). How will the potential global distribution areas of *L. erysimi* and *E. corollae* change under future climates? What are the key environmental factors for the survival and dispersal of both? Is it possible to introduce the *E. corollae* for biological control in areas with serious *L. erysimi* damage? These questions are not yet clear. Therefore, we used the MaxEnt model and Arc GIS to construct an ecological niche model to analyze the potential distribution areas of *L. erysimi* and its natural enemy, *E. corollae* under current (1970-2000) and future climates (2041-2060) in our study to clarify the weights of different environmental variables on the ecological distribution of both, and to determine the key environmental parameters for the survival and dispersal of both. This study will provide theoretical and data support for the monitoring and early warning of *L. erysimi* and its biological control.

## Materials and methods

### Collection and screening of geographic distribution data

A total of 707 *L. erysimi* and 26173 *E. corollae* geographic coordinates of historical occurrence were obtained by visiting the GBIF (https://www.gbif.org/) (Beck et al., 2014) and CABI (https://www.cabi.org/) (Pasiecznik et al., 2005) websites. To avoid overfitting, we created a 2 km × 2 km raster, took only one coordinate point data in each raster, and removed duplicate points and coordinate points on the sea surface (Welch and Harwood, 2014). Finally, we filtered 570 *L. erysimi* and 9775 *E. corollae* coordinates data and saved them in “.CSV” format.

### Climate data acquisition and screening

**Figure 1:**
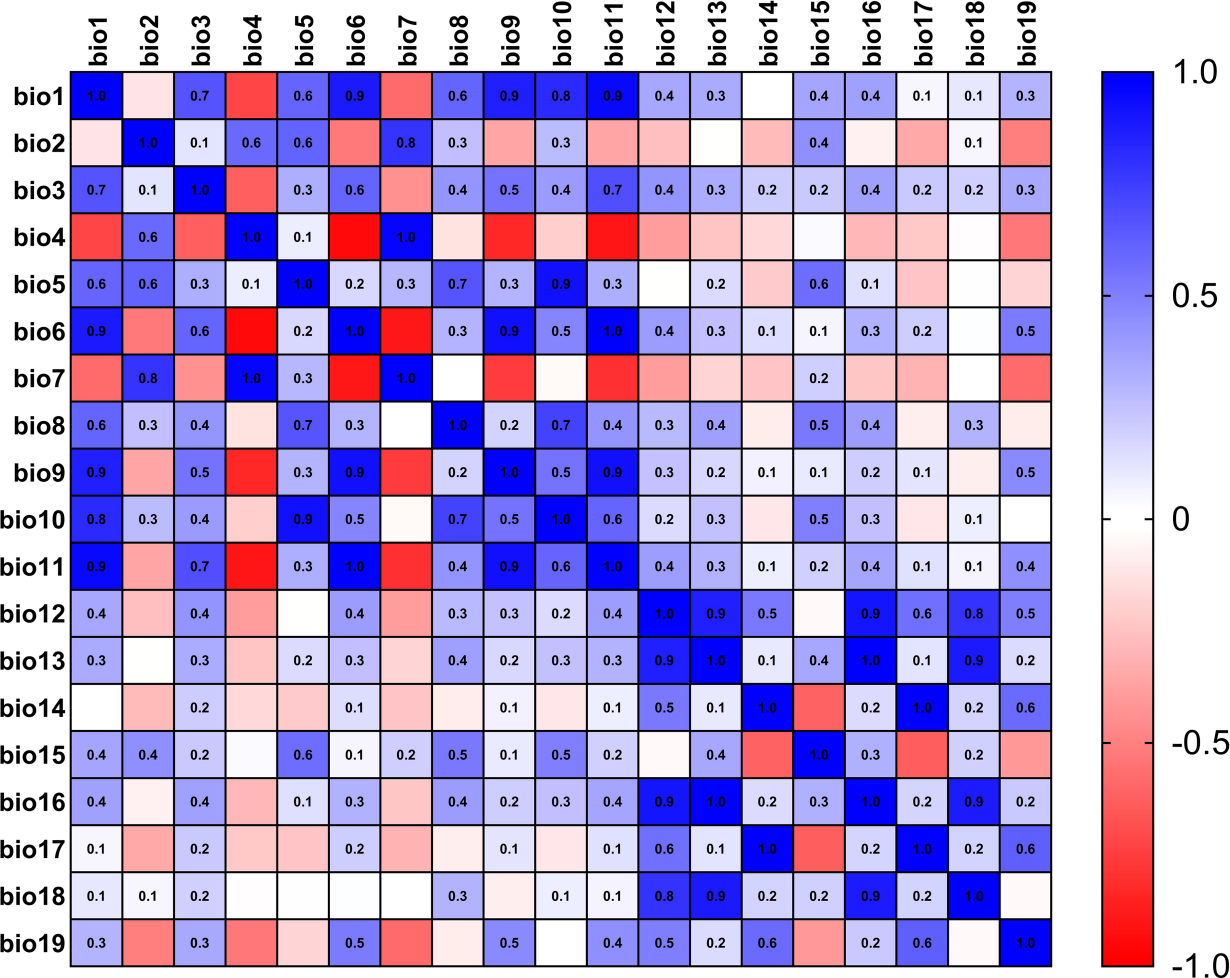
Heat map for correlation analysis of environmental factors in *L. erysimi*.

**Figure 2:**
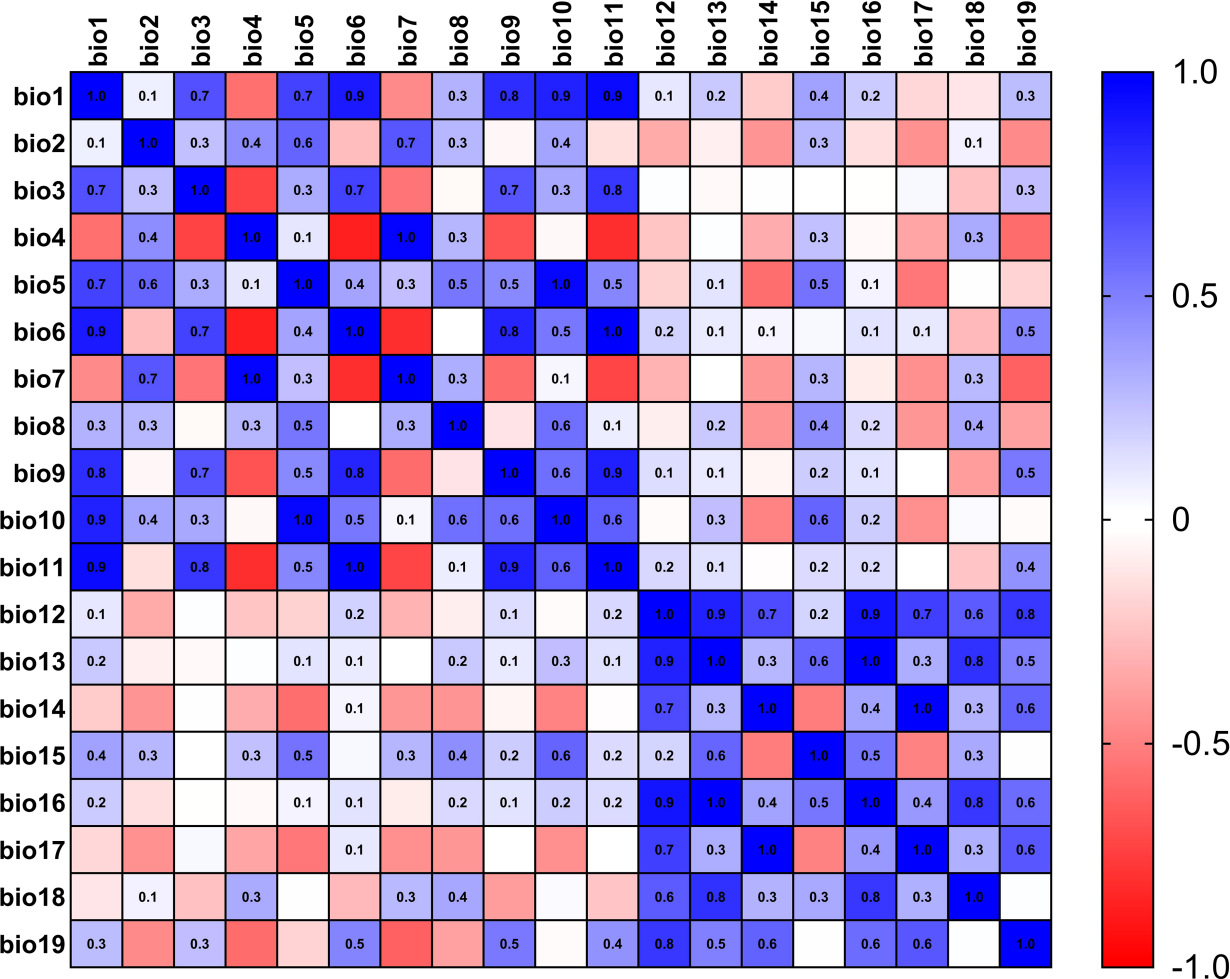
Heat map for correlation analysis of environmental factors in *E. corollae*.

The current 19 environmental variables data (**Table 1**) used in this study were obtained from the WorldClim (https://www.worldclim.org) (Fick and Hijmans, 2017) database, which was released in January 2020 and spans the period 1970-2000 with a precision of 2.5 arc-minutes. Using the knife cut method in MaxEnt 3.4.4 (Phillips and Dudík, 2008) software to rank the contribution of environmental factors, and then using SPSS 26.0 to analyze the correlation between environmental factors, when the absolute value of the correlation between two ecological factors was greater than or equal to 0.8, only one representative environmental factor will be kept (Li et al., 2022), meanwhile, the heat map for correlation analysis of environmental factors in *L. erysimi* (**Figure 1**) and *E. corollae* (**Figure 2**) was constructed. Finally, we removed the nine environmental variables factors for the *L. erysimi* (Bioclimatic (Bio) 6, Bio7, Bio9, Bio10, Bio11, Bio13, Bio14, Bio16) and the nine environmental variables for the *E. corollae* (Bio4, Bio7, Bio9, Bio10, Bio11, Bio12, Bio13, Bio17), and obtained 11 *L. erysimi* (Bio1, Bio2, Bio3, Bio4, Bio5, Bio8, Bio12, Bio15, Bio17, Bio18, Bio19) and 11 environmental variable factors of *E. corollae* (Bio1, Bio2, Bio3, Bio5, Bio6, Bio8, Bio14, Bio15, Bio16, Bio18, Bio19).

**Table 1:** Climate and environment variables.

Variables	Bioclimatic variables	Original resolutions	Online sources
Bio1	Annual average temperature (°C)	
Bio2	Monthly mean temperature difference between day and night (°C)	
Bio3	Ratio of diurnal temperature difference to annual temperature difference (%)	
Bio4	Seasonal variance of temperature (°C)	
Bio5	Maximum temperature in the warmest month (°C)	
Bio6	Lowest temperature in the coldest month (°C)	
Bio7	Annual variation range of temperature (°C)	
Bio8	Average temperature in the wettest quarter (°C)
Bio9	Average temperature in the driest quarter (°C)	2.5 minutes	WorldClim
Bio10	Average temperature of the warmest quarter (°C)		
Bio11	Average temperature in the coldest quarter (°C)		
Bio12	Average annual precipitation (mm)		
Bio13	Precipitation in the wettest month (mm)		
Bio14	Precipitation in the driest month (mm)		
Bio15	Seasonal variation coefficient of precipitation (%)		
Bio16	Precipitation in the wettest quarter (mm)		
Bio17	Precipitation in the driest quarter (mm)		
Bio18	Precipitation in the warmest quarter (mm)		
Bio19	Precipitation in the coldest quarter (mm)		

In this study, environmental date from three GHG emission scenarios, Representative Concentration Pathway (RCP) 2.6, RCP4.5 and RCP8.5, were selected to project the future climate suitability areas for the *L. erysimi* and *E. corollae* under future climate conditions. Three future scenarios were obtained from the WorldClim. These bioclimatic variables ran in the model were selected from Coupled Model Intercomparison Project Phase 6 (CMIP6), and their accuracy was 2.5 arc-minutes. RCP 2.6 reveals carbon dioxide emissions could peak globally in 2020 and start to decline in 2080. It also shows that the atmospheric concentration would peak in the middle of the century, followed by a steady decline. In the RCP 4.5, emissions will peak in the middle of the century and then rapidly decline over the next 30 years. The emissions will stabilize to be half of what the levels were in the year 2000. Carbon dioxide concentration will continue to increase according to current trends, but will stabilize and the rate of increased emissions will not be as rapid as previously expected. Serving as the antithesis of RCP 2.6, RCP 8.5 serves as the worst case scenario for the future of emissions. In this RCP, emissions continue to drastically increase throughout the century – predominantly during early and middle parts of the current century. By 2100, the emissions will have stabilized, but will rest at 30 gigatonnes of carbon as opposed to the eight gigatonnes in 2000 (Van Vuuren et al., 2011).

### Model construction and evaluation

First, we imported the filtered bioclimatic variable data (1970-2000) for the current GHG emission scenario into the MaxEnt model, and then imported the geographic distribution data of *L. erysimi* and *E. corollae* into the model separately, and randomly selected 75% of the data as the training set for the experiment, and the remaining 25% of the coordinate data as the test set, with a number of iterations of 10. MaxEnt software settings are as follows: check “Create response curves” and “Do jackknife to measure variable importance”; “out format” option select “Logistic”, check “Random seed”; set “Random test percentage” to 25, check “Write plot data”, the rest keep the default settings. The raster files were reclassified using the Spatial Analyst option of the Arc toolbox in ArcMap 10.8 software, and the distribution areas were set to four gradients (Welch and Harwood, 2014): Unsuitable area (0-0.2), Low suitable area (0.2-0.4), Moderately suitable area (0.4-0.6), and Highly suitable area (0.6-1).

In this study, the area under the receiver operating characteristic curve (AUC) (Fielding and Bell, 1997) was used as a measure of model prediction accuracy and the interval ranges from 0.5 to 1. 0.5 corresponds to a completely random prediction, in the range of 0.5-0.7 indicates poor accuracy of the prediction results, in the range of 0.7-0.9 indicates moderate accuracy of the prediction results, and when the prediction results are greater than 0.9, it indicates very high accuracy of the prediction results (Barry and Elith, 2006).

## 3 Results

### Model performance evaluation

The MaxEnt model was constructed using the geographic distribution data of *L. erysimi* and *E. corolla* and the screened environmental variable data, and the performance of the MaxEnt model was evaluated using the mean values of AUCs obtained after 10 iterations of the model. The mean values of all AUCs were calculated to be greater than or equal to 0.8 (**Table 2**), which indicated that the model had a good performance and could predict the potential distribution areas of *L. erysimi* and *E. corolla* more accurately.

**Table 2:** Area under the receiver operating characteristic curve.

Species	AUC	Current	2050s		
			RCP2.6	RCP4.5	RCP8.5
*Lipaphis erysimi*	Testing data	0.886	0.883	0.883	0.885
*Eupeodes corollae* Fabricius	Testing data	0.802	0.800	0.802	0.801

### Potential distribution area changes of *L. erysimi* under current and future climate conditions

**Figure 3:**
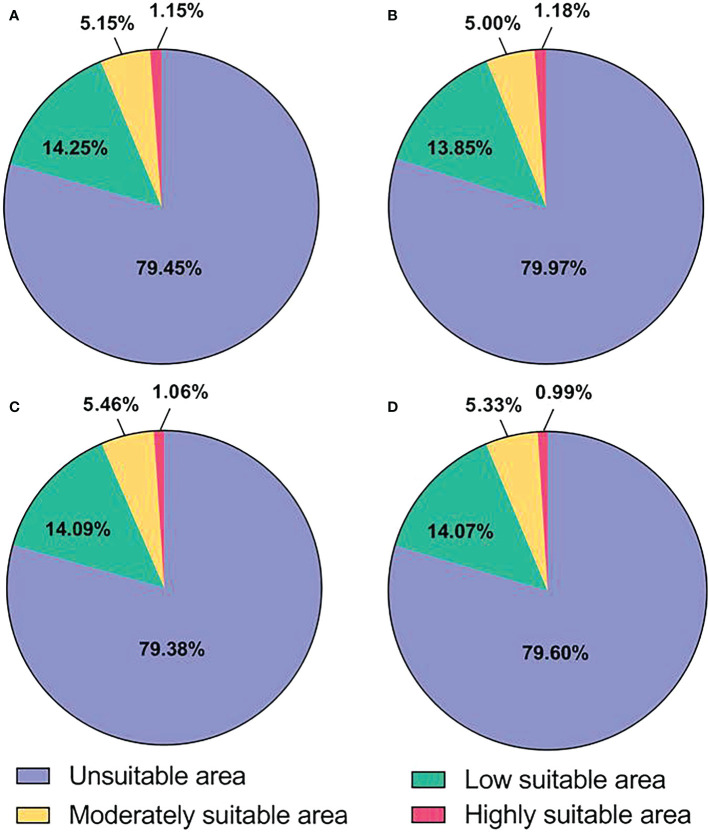
Proportion of suitable area for *L. erysimi*. **(A)**, Current climatic conditions. **(B)**, RCP2.6 climatic conditions. **(C)**, RCP4.5 climatic conditions. **(D)**, RCP8.5 climatic conditions.

The potential distribution areas of *L. erysimi* under current and future climatic conditions are shown in **Figure 3** and **Table 3**. Under current climatic conditions, the highly suitable area of *L. erysimi* is mainly distributed in western Europe, China Taiwan, southern coastal areas of China, eastern United States, northern Myanmar, and northern India, covering 1.67 × 10^5^ km^2^ or 1.15% of the total area; The moderately suitable area is mainly distributed in southwestern Europe, eastern and southern China, eastern United States, southeastern South America, and northern India, with an area of 7.48 × 10^5^ km^2^, accounting for 5.15% of the total area; The low suitable area is widely distributed in Europe, America, Africa, Asia, and Australia, accounting for 14.25% of the total area. Under the RCP2.6, the area of highly suitable area of *L. erysimi* increased, the area of moderate and low suitable area decreased, and the total suitable area decreased by 7.58 × 10^4^ km^2^. Under both the RCP4.5 and RCP8.5, the area of high and low suitable area of *L. erysimi* decreased, while the area of moderately suitable area increased, and the total suitable area increased by 9.10 × 10^3^ km^2^ under the RCP4.5 and decreased by 2.22 × 10^4^ km^2^ under the RCP8.5. This indicates that the expansion of *L. erysimi* will be inhibited under the RCP8.5, while the expansion of *L. erysimi* will be promoted under the RCP2.6 and RCP4.5.

**Table 3:** The regions and area of suitable area for *L. erysimi*.

	Highly suitable area	Moderately suitable area	Low suitable area
Current (Total area is 2.98× 10^6^ km^2^)	Eastern United States, Eastern Brazil, Eastern Uruguay, Western Germany, Western Netherlands, Western Denmark, Ireland, United Kingdom, France, Western Georgia, Belgium, Northern India, Northern Myanmar, Eastern Coast of Vietnam, Southern Coast of China, China Taiwan, Korea, Southern Japan (1.67×10^5^ km^2^)	Eastern United States, Eastern Brazil, Eastern Argentina, Eastern Germany, Eastern Netherlands, Eastern Denmark, Western Poland, Western Ukraine, Uruguay, Southern Sweden, Northern Italy, United Kingdom, France, Czech Republic, Hungary, Romania, Bulgaria, Northern Turkey, Georgia, Northern India, Nepal, Northern Myanmar, Bangladesh, Vietnam, Southeastern China, North Korea, South Korea, Japan, Southeastern Australia, Northern New Zealand (7.48×10^5^ km^2^)	Southern North America, Northern and Eastern South America, Southern and Eastern Africa, Western and Central Europe, Southeastern Asia, Eastern Australia (2.07×10^6^ km^2^)
RCP2.6 (Total area is 2.91× 10^6^ km^2^)	Eastern United States, Eastern Brazil, Eastern Uruguay, Western Germany, Western Netherlands, Western Denmark, Ireland, United Kingdom, France, Western Georgia, Belgium, Northern India, Northern Myanmar, Eastern Coast of Vietnam, Southern Coast of China, China Taiwan, Southern Japan (1.71×10^5^ km^2^)	Eastern United States, Eastern Brazil, Eastern Argentina, Eastern Germany, Eastern Netherlands, Eastern Denmark, Western Poland, Western Ukraine, Uruguay, Southern Sweden, Northern Italy, United Kingdom, France, Czech Republic, Hungary, Romania, Bulgaria, Northern Turkey, Georgia, Northern India, Nepal, Northern Myanmar, Bangladesh, Vietnam, Southern China Coast, South Korea, Japan, Southeastern Australia, Northern New Zealand (7.26×10^5^ km^2^)	Southern North America, Northern and Eastern South America, Southern and Eastern Africa, Western and Central Europe, Southeastern Asia, Eastern Australia (2.01×10^6^ km^2^)
RCP4.5 (Total area is 3.00× 10^6^ km^2^)	Eastern United States, Eastern Brazil, Eastern Uruguay, Western Germany, Western Netherlands, Western Denmark, Ireland, United Kingdom, France, Belgium, Northern India, Northern Myanmar, Eastern Coast of Vietnam, Southern Coast of China, China Taiwan, Southern Japan (1.53×10^5^ km^2^)	Eastern United States, Eastern Brazil, Eastern Argentina, Eastern Germany, Eastern Netherlands, Eastern Denmark, Western Poland, Western Ukraine, Uruguay, Southern Sweden, Northern Italy, United Kingdom, France, Czech Republic, Hungary, Romania, Bulgaria, Northern Turkey, Georgia, Northern India, Nepal, Northern Myanmar, Vietnam, Southeastern China, North Korea, South Korea, Japan, Southeastern Australia, Northern New Zealand (7.93×10^5^ km^2^)	Southern North America, Northern and Eastern South America, Southern and Eastern Africa, Western and Central Europe, Southeastern Asia, Eastern Australia (2.04×10^6^ km^2^)
RCP8.5 (Total area is 2.96× 10^6^ km^2^)	Eastern United States, Eastern Brazil, Western Germany, Western Netherlands, Western Denmark, Ireland, United Kingdom, Southeastern France, Belgium, Northern India, Northern Myanmar, Eastern Coast of Vietnam, Southern Coast of China, China Taiwan, Southwestern Korea, Southern Japan (1.44×10^5^ km^2^)	Eastern United States, Eastern Brazil, Eastern Argentina, Eastern Germany, Eastern Netherlands, Eastern Denmark, Western Poland, Western Ukraine, Uruguay, Southern Sweden, Northern Italy, United Kingdom, France, Czech Republic, Hungary, Romania, Bulgaria, Northern Turkey, Georgia, Northern India, Nepal, Northern Myanmar, Vietnam, Southeastern China, North Korea, South Korea, Japan, Southeastern Australia, Northern New Zealand (7.74×10^5^ km^2^)	Southern North America, Northern and Eastern South America, Southern and Eastern Africa, Western and Central Europe, Southeastern Asia, Eastern Australia (2.04×10^6^ km^2^)

### Changes in the potential distribution area of *E. corollae* under current and future climatic conditions

**Figure 4:**
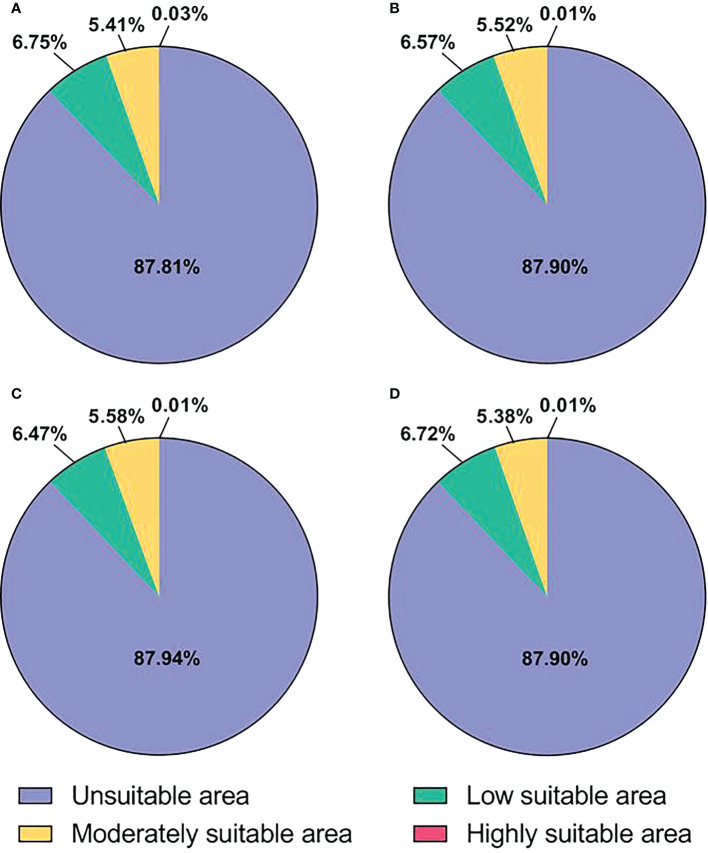
Proportion of suitable area for *E*. *corollae*. **(A)**, Current climatic conditions. **(B)**, RCP2.6 climatic conditions. **(C)**, RCP4.5 climatic conditions. **(D)**, RCP8.5 climatic conditions.

The potential distribution areas of *E. corollae* under current and future climatic conditions are shown in **Figure 4** and **Table 4**. Under current climatic conditions, the highly suitable area of *E. corollae* is mainly distributed in China Taiwan, northern India, and northern Myanmar, with an area of 3.69×10^3^ km^2^, accounting for 0.03% of the total area; The moderately suitable area is mainly distributed in central Europe and eastern Asia, with scattered distribution in coastal areas of America, with an area of 7.85×10^5^ km^2^, accounting for 5.41% of the total area; The low suitable area is distributed in northeastern Europe, western North America, southern South America, southeastern China, eastern United States, and southern Australia, with an area of 9.80×10^5^ km^2^, accounting for 6.75% of the total area. Under all three future climatic conditions, the area of the highly suitable area of *E. corollae* was significantly reduced (by more than 65%) compared with the current climatic conditions, and the total suitable area was reduced by 1.29×10^4^ km^2^, 1.78×10^4^ km^2^ and 1.25×10^4^ km^2^, indicating that all three future climatic conditions were unfavorable for the expansion of *E. corollae*.

**Table 4:** The regions and area of suitable area for *E. corollae*.

	Highly suitable area	Moderately suitable area	Low suitable area
Current (Total area is 1.76×10^6^ km^2^)	Northern India, Eastern Nepal, Northern Myanmar, China Taiwan, China, Southern Japan (3.69×10^3^ km^2^)	Eastern United States, Western Canada, Southern Chile, Iceland, Ireland, United Kingdom, France, Germany, Belgium, Netherlands, Denmark, Poland, Ukraine, Georgia, Czech Republic, Hungary, Romania, Bulgaria, Greece, Italy, Norway, Southern Sweden, Southern Finland, Northern Spain, Northern Portugal, Northern India, Nepal, Northern Myanmar, Southeastern China, South Korea, Japan (7.85×10^5^ km^2^)	Northwestern and Southeastern North America, Southern South America, Eastern and Southern Europe, Southeastern Asia, Southern Australia (9.80×10^5^ km^2^)
RCP2.6 (Total area is 1.75×10^6^ km^2^)	Northern India, Northern Myanmar, Central Japan (1.13×10^3^ km^2^)	Eastern United States, Western Canada, Southern Chile, Iceland, Ireland, United Kingdom, France, Germany, Belgium, Netherlands, Denmark, Poland, Ukraine, Georgia, Czech Republic, Hungary, Romania, Bulgaria, Greece, Italy, Norway, Southern Sweden, Southern Finland, Northern Spain, Northern Portugal, Northern India, Nepal, Northern Myanmar, Southeastern China, South Korea, Japan (8.01×10^5^ km^2^)	Northwestern and Southeastern North America, Southern South America, Eastern and Southern Europe, Southeastern Asia, Southern Australia (9.53×10^5^ km^2^)
RCP4.5 (Total area is 1.75×10^6^ km^2^)	Northern India, Northern Myanmar, Central Japan (1.13×10^3^ km^2^)	Eastern United States, Western Canada, Southern Chile, Iceland, Ireland, United Kingdom, France, Germany, Belgium, Netherlands, Denmark, Poland, Ukraine, Georgia, Czech Republic, Hungary, Romania, Bulgaria, Greece, Italy, Norway, Southern Sweden, Southern Finland, Northern Spain, Northern Portugal, Northern India, Nepal, Northern Myanmar, Southeastern China, South Korea, Japan (8.10×10^5^ km^2^)	Northwestern and Southeastern North America, Southern South America, Eastern and Southern Europe, Southeastern Asia, Southern Australia (9.39×10^5^ km^2^)
RCP8.5 (Total area is 1.76×10^6^ km^2^)	Northern India, Northern Myanmar, Central Japan (1.26×10^3^ km^2^)	Eastern United States, Western Canada, Southern Chile, Iceland, Ireland, United Kingdom, France, Germany, Belgium, Netherlands, Denmark, Poland, Ukraine, Georgia, Czech Republic, Hungary, Romania, Bulgaria, Greece, Italy, Norway, Southern Sweden, Southern Finland, Northern Spain, Northern Portugal, Northern India, Nepal, Northern Myanmar, Southeastern China, South Korea, Japan (7.80×10^5^ km^2^)	Northwestern and Southeastern North America, Southern South America, Eastern and Southern Europe, Southeastern Asia, Southern Australia (9.74×10^5^ km^2^)

### Influence of environmental variables on the distribution of *L. erysimi* and *E. corollae*

**Figure 5:**
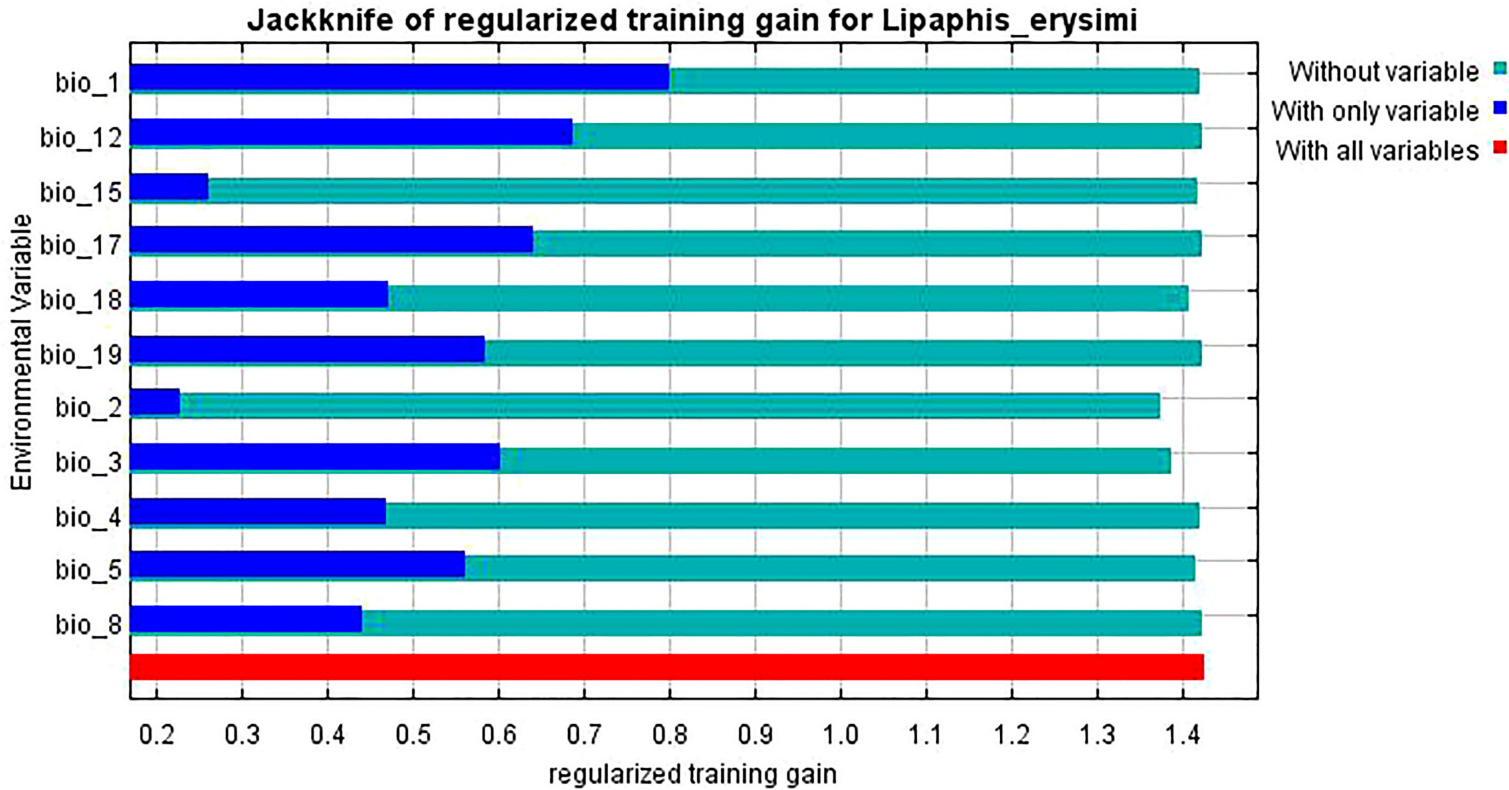
Evaluation of environmental variables affecting the distribution of *L. erysimi* by Jackknife test.

**Figure 6:**
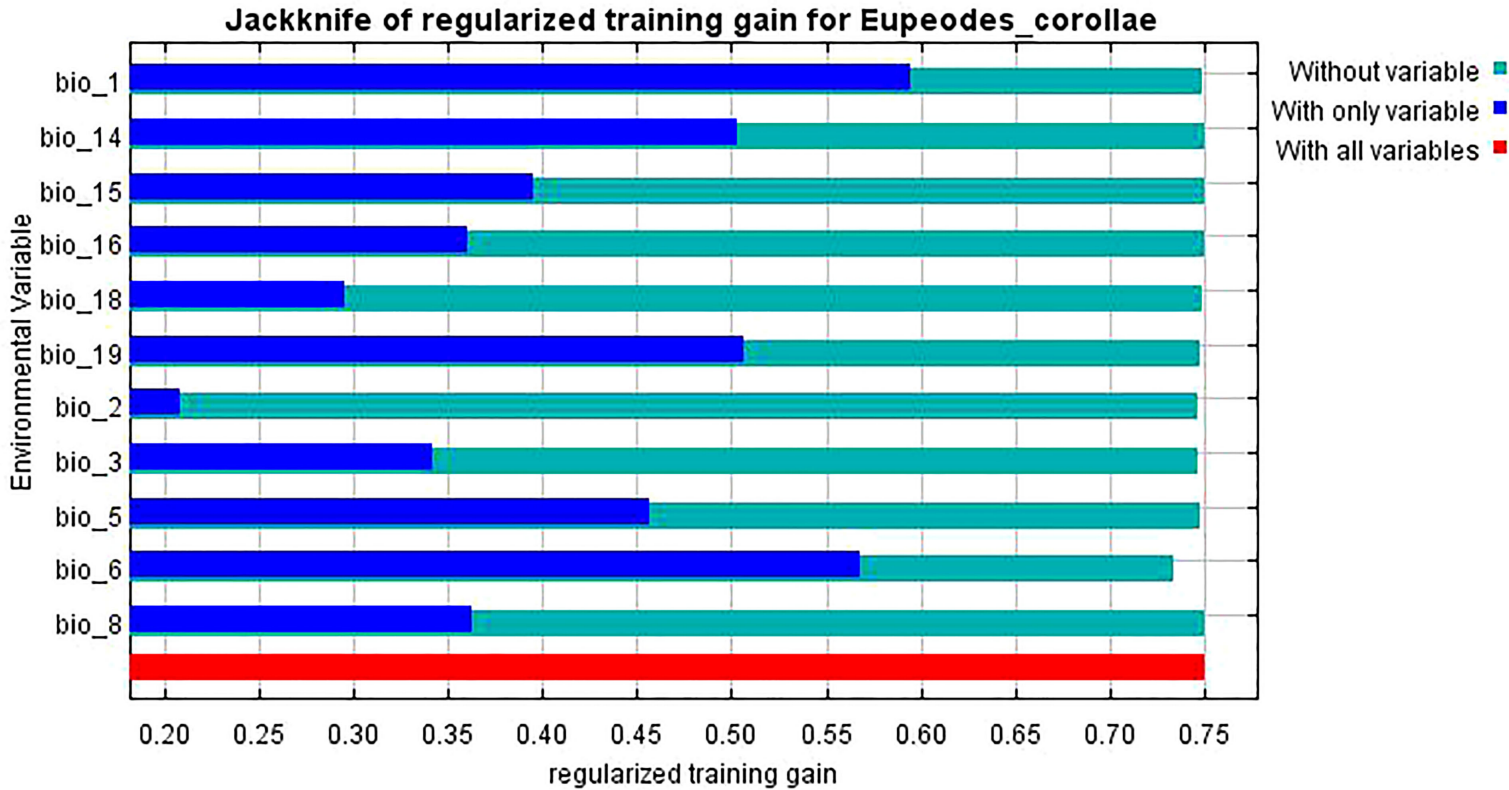
Evaluation of environmental variables affecting the distribution of *E. corollae* by Jackknife test.

The knife-cut test can show the magnitude of the contribution of environmental variables to the gain in the distribution of *L. erysimi* and *E. corollae*, and the correlation analysis of environmental variables using the knife-cut method yielded the results of the effect of environmental variables on the distribution of *L. erysimi* (**Figure 5**) and *E. corollae* (**Figure 6**). In *L. erysimi*, the model gains of Bio1 (Annual average temperature) and Bio12 (Annual average precipitation) were 0.8 and 0.68, respectively, with high regularization gains, which contributed more to the distribution gain of *L. erysimi*. In *E. corollae*, Bio1 (Annual average temperature) and Bio6 (Lowest temperature in the coldest month) had the highest model gains of 0.59 and 0.56, respectively, indicating that these two variables had the greatest influence on the distribution of *E. corolla.*

**Figure 7:**
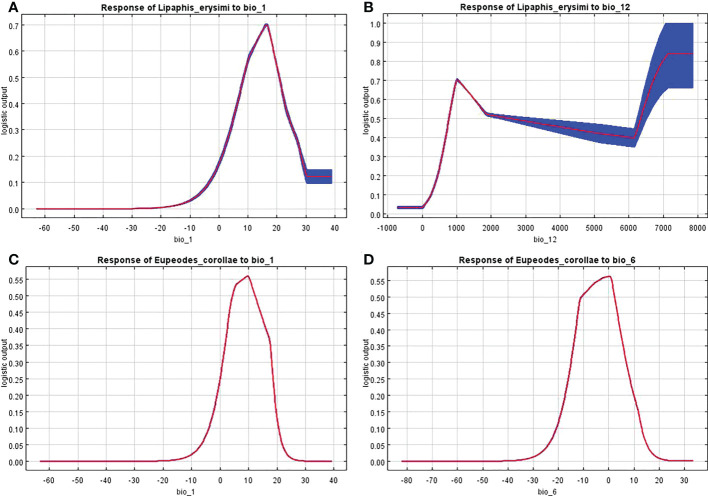
Response curve of distribution probability of *L. erysimi* and *E*. *corollae* to bioclimatic factors. **(A)**, Response curve of *L. erysimi* to Bio1. **(B)**, Response curve of *L. erysimi* to Bio12. **(C)**, Response curve of *E. corollae* to Bio1. **(D)**, Response curve of *E. corollae* to Bio6.

The response curves of the dominant environmental variables showed that *L. erysimi* had no suitableness when the annual average temperature was below 0°C and above 29°C, and its suitableness was highest when the annual average temperature was around 18°C (**Figure 7A**). The highest suitableness was found when the annual average precipitation reached 1000 mm, and then declined with increasing annual average precipitation. Since the standard error of the response curve is large when the annual average precipitation more than 4000 mm, it does not be consider (**Figure 7B**). The response curves of the annual average temperature and the lowest temperature in the coldest month were consistent in the *E. corolla*, both increasing and then decreasing, with the highest suitableness at the annual average temperature around 10°C (**Figure 7C**) and the highest suitableness at the lowest temperature in the coldest month around 0°C (**Figure 7D**).

The curves show the mean response of the 10 replicate MaxEnt runs. Red indicates the average value and blue indicates ± SD.

## Discussion

The highly and moderately suitable areas of *L. erysimi* under current climatic conditions in our study were mainly distributed at 15°-65°N and 15°-55°S, and are distributed in the Americas, Europe, Asia, and Australia, with concentrations in China, the United States, France, India, and so on, which is generally consistent with the reported distribution of *L. erysimi* (Patel et al., 2004; Desneux et al., 2006; Adhab and Schoelz, 2015; Bai et al., 2022). It proves the reliability of the prediction software used in our study. The area of highly suitable area of *L. erysimi* increased in the United States and India and decreases in Korea under the RCP2.6. The area of highly suitable area of *L. erysimi* increased in India and decreased in the United States and Korea under the RCP4.5. The area of highly suitable area of *L. erysimi* in France was reduced under the RCP8.5. The range and area of highly suitable area of *E. corollae* under the current climate conditions are smaller than that of *L. erysimi*, mainly distributed in 23.5-65°N and 30-55°S. The area of highly suitable area of *E. corollae* decreases remarkably under the three future climate conditions. China Taiwan, changes from a highly suitable area to a moderately suitable area, and the area of highly suitable area in northern India and northern Myanmar decreases. India is the third largest producer of rapeseed mustard, accounting for 19.8% of global acreage and 9.8% of total production, and damage by *L. erysimi* can result in up to 90% yield loss (Palial et al., 2022). Most of India is suitable for *L. erysimi* under current climatic conditions, and the suitable area for *L. erysimi* in India will increase under the RCP2.6 and RCP4.5. Whereas, *E. corollae* only be suitable in a small area in northern India, and its suitable area will decrease in future climatic conditions, which may increase the difficulty of using *E. corollae* for biological control of *L. erysimi* in India. Zhang et al. (2022a) Predicting the distribution of the Asian Longhorned Beetle, *Anoplophora glabripennis* (Coleoptera: Cerambycidae) and its natural enemies in China. The results showed that the Northern China (e.g., Xinjiang, Gansu, and Inner Mongolia), where *A. glabripennis* causes more serious damage, is also a potential suitable area for *Dastarcus helophoroides* and *Dastarcus major*, which provides a potential strategy for the management of *A. glabripennis*. In our study, the suitable area of *E. corollae* in Western Europe, southern China, eastern United States, Japan, and Korea partially overlap with those of the *L. erysimi*, it is speculated that the use of *E. corollae* for *L. erysimi* control in these areas may present a good effect.

Environmental variables act on species distributions at spatial scales that vary in size, and at relatively large scales, species interactions are often weakened and climatic variables play a major role (Hortal et al., 2010). Insects are poikilotherm, and temperature is crucial to their development. The distribution of insects and the number of generations in different regions can be inferred by measuring the developmental temperature and effective accumulation temperature of insects. In addition, humidity is closely related to insect life activities such as pupation, fledging, and evaporation of water from insects (Eberle et al., 2022). The two most critical environmental variables affecting the potential geographic distribution of *L. erysimi* in our study were Bio1 (Annual average temperature) and Bio12 (Annual average precipitation), and the two most critical environmental variables affecting the potential geographic distribution of *E. corollae* were Bio1 (Annual average temperature) and Bio6 (Lowest temperature in the coldest month). The suitableness of *L. erysimi* was the highest when the annual average temperature was 10°C-20°C. The suitableness of *L. erysimi* was the highest when the annual average precipitation was 1000 mm, and then decrease with the increase of annual average precipitation. Studies have shown that the optimum temperature of *L. erysimi* is at or below 20°C. Cloudy and cold weather is helpful to the development of *L. erysimi* (Singh et al., 2007). The occurrence of *L. erysimi* is severe in dry weather and the number of *L. erysimi* is inversely proportional to relative humidity when humidity is high (Mishra and Kanwat, 2018), the predicted results of our study are consistent with this result. The two most critical environmental variables influencing the distribution of *E. corollae* are both temperatures, with the highest suitableness at the annual average temperature of 10°C and the lowest temperature in the coldest month of 0°C, and *E. corollae* has been found to maintain egg production and predation at temperatures as low as 12°C (Moerkens et al., 2021), but no studies on the predatory performance of *E. corollae* at low temperatures have been reported, and it is speculated that conditions of 10°C-15°C may be conducive to the predation, development, and reproduction of *E. corollae*.

The MaxEnt ecological niche model has some limitations in that it only analyzes the influence of abiotic factors on species distribution, while in reality species distribution is also influenced by biotic factors (Wang et al., 2019). *L. erysimi* often follows seedlings dispersed by anthropogenic transfer, thus the actual distribution range is larger than predicted (Zhao et al., 2022a). In addition, if there is excessive humidity in the field or continuous rainfall, the aphids will be washed away and die in large numbers by the rain, leading to a reduction in the population density of *E. corollae* due to lack of food (Putra and Yasuda, 2006).

In conclusion, this study used the geographic distribution data of the historical occurrence of *L. erysimi* and *E. corollae* to project their potential distribution areas under current and future climates based on the MaxEnt model, and analyzed the key environmental factors affecting the survival and spread of both, concluding that RCP2.6 and RCP4.5 will be favorable for the survival and spread of *L. erysimi*, but will inhibit the spread of the natural enemy *E. corolla*; Low temperatures favor the predation of *E. corollae* but also promote the occurrence of *L. erysimi*; High humidity reduces population densities of *L. erysimi* and *E. corolla*; Therefore, we should fully consider the temperature and humidity in the field when using *E. corolla* to control *L. erysimi* and develop a scientific and reasonable control strategy. This study can provide theoretical and data support for the monitoring and early warning and biological control of *L. erysimi*.

## Data availability statement

The original contributions presented in the study are included in the article/supplementary material. Further inquiries can be directed to the corresponding author.

## Author contributions

YL, AW, SP, JJ, XY and SZ participated in the study design and analysis of the manuscript. JL, SY and RZ participated in the study design and helped to draft the manuscript. Supervision and financial support by SZ, revised and processed. All authors contributed to the article and approved the submitted version.

## Funding

This study was supported by Hainan Major Science and Technology Project (ZDKJ201901). Hainan Province Science and Technology Special Fund (ZDYF2022XDNY163).

## Conflict of interest

The authors declare that the research was conducted in the absence of any commercial or financial relationships that could be construed as a potential conflict of interest.

## Publisher’s note

All claims expressed in this article are solely those of the authors and do not necessarily represent those of their affiliated organizations, or those of the publisher, the editors and the reviewers. Any product that may be evaluated in this article, or claim that may be made by its manufacturer, is not guaranteed or endorsed by the publisher.

## References

[B1] Adhab M. A. Schoelz J. E. (2015). Report of the turnip aphid, *Lipaphis erysimi* (Kaltenbach 1843) from Missouri, USA. J. Plant Prot. Sci. 55, 327–328. doi: 10.1515/jppr-2015-0035

[B2] Bai P. H. Hu R. R. Bian D. B. Liu B. S. Li N. Hu C. F. (2022). Laboratory toxicity evaluation of *Vitex negundo* extracts on *Lipaphis erysimi* . Tianjin Agric. Sci. 28, 57–60. doi: 10.3969/j.issn.1006-6500.2022.S.014

[B3] Barreca A. I. (2012). Climate change, humidity, and mortality in the united states. J. Environ. Econ Manage. 63, 19–34. doi: 10.1016/j.jeem.2011.07.004 25328254 PMC4199665

[B4] Barry S. Elith J. (2006). Error and uncertainty in habitat models. J. Appl. Ecol. 43, 413–423. doi: 10.1111/j.1365-2664.2006.01136.x

[B5] Beck J. Böller M. Erhardt A. Schwanghart W. (2014). Spatial bias in the GBIF database and its effect on modeling species' geographic distributions. Ecol. Inform. 19, 10–15. doi: 10.1016/j.ecoinf.2013.11.002

[B6] Chattopadhyay C. Agrawal R. Kumar A. Singh Y. P. Roy S. K. Khan S. A.. (2005). Forecasting of *Lipaphis erysimi* on oilseed brassicas in India–a case study. Crop Prot. 24, 1042–1053. doi: 10.1016/j.cropro.2005.02.010

[B7] Davis J. J. (1914). New or little known species of aphididae. Can. Entomol. 46, 226–236. doi: 10.4039/Ent46226-7

[B8] Desneux N. Rabasse J. M. Ballanger Y. Kaiser L. (2006). Parasitism of canola aphids in France in autumn. J. Pest Sci. 79, 95–102. doi: 10.1007/s10340-006-0121-1

[B9] Dong K. Dong Y. Luo Y. Z. (2004). Studies on the developmental zero temperature and effective accumulated temperature of *Syrphus corollae* fabricius. J. Yunnan Agric. Univ. 19, 177–178+198. doi: 10.16211/j.issn.1004-390x(n).2004.02.013

[B10] Dong Z. He Y. Ren Y. Wang G. Chu D. (2022). Seasonal and year-round distributions of *Bactrocera dorsalis* (Hendel) and its risk to temperate fruits under climate change. Insects 13, 550. doi: 10.3390/insects13060550 35735887 PMC9225012

[B11] Eberle S. Schaden L. M. Tintner J. Stauffer C. Schebeck M. (2022). Effect of temperature and photoperiod on development, survival, and growth rate of mealworms, *Tenebrio molitor* . Insects 13, 321. doi: 10.3390/insects13040321 35447763 PMC9029539

[B12] Elith J. Phillips S. J. Hastie T. Dudík M. Chee Y. E. Yates C. J. (2011). A statistical explanation of MaxEnt for ecologists. Divers. Distrib. 17, 43–57. doi: 10.1111/j.1472-4642.2010.00725.x

[B13] Fick S. E. Hijmans R. J. (2017). WorldClim 2: new 1-km spatial resolution climate surfaces for global land areas. Int. J. climatol. 37, 4302–4315. doi: 10.1002/joc.5086

[B14] Fielding A. H. Bell J. F. (1997). A review of methods for the assessment of prediction errors in conservation presence/absence models. Environ. Conserv. 24, 38–49. doi: 10.1017/S0376892997000088

[B15] He J. L. Sun X. Q. Gui L. M. Ye W. J. (1990). A preliminary study on the biology of *Eupeodes corollae* fabricius in shanghai. J. Shanghai Agric. Univ. 8, 221–228+234.

[B16] Hortal J. Roura- Pascual N. Sanders N. J. Rahbek C. (2010). Understanding (insect) species distributions across spatial scales. Ecography. 33, 51–53. doi: 10.1111/j.1600-0587.2009.06428.x

[B17] Khaliq A. M. Javed M. Sohail M. Sagheer M. (2014). Environmental effects on insects and their population dynamics. J. Entomol. Zool Stud. 2, 1–7.

[B18] Koramutla M. K. Aminedi R. Bhattacharya R. (2016). Comprehensive evaluation of candidate reference genes for qRT-PCR studies of gene expression in mustard aphid, *Lipaphis erysimi* (Kalt). Sci. Rep. 6, 1–10. doi: 10.1038/srep25883 27165720 PMC4863174

[B19] Li Z. Liu Y. Zeng H. (2022). Application of the MaxEnt model in improving the accuracy of ecological red line identification: A case study of zhanjiang, China. Ecol. Indic. 137, 108767. doi: 10.1016/j.ecolind.2022.108767

[B20] Li G. T. Zhang F. S. Gao J. F. (1996). Observation on the biological characteristics of *Eupeodes corollae* fabricius. J. Jilin Agric. Univ. 18, 154–157. doi: 10.13327/j.jjlau.1996.s1.051

[B21] Liu Y. Zhou K. Xia Q. (2018). A MaxEnt model for mineral prospectivity mapping. Nat. Resour. Res. 27, 299–313. doi: 10.1007/s11053-017-9355-2

[B22] Mishra S. K. Kanwat P. M. (2018). Seasonal incidence of mustard aphid, *Lipaphis erysimi* (Kalt) and its major predator on mustard and their correlation with abiotic factors. J. Entomol. Zool Stud. 6, 831–836.

[B23] Moerkens R. Boonen S. Wäckers F. L. Pekas A. (2021). Aphidophagous hoverflies reduce foxglove aphid infestations and improve seed set and fruit yield in sweet pepper. Pest Manage. Sci. 77, 2690–2696. doi: 10.1002/ps.6342 33638225

[B24] Palial S. Kumar S. Atri C. Sharma S. Banga S. S. (2022). Antixenosis and antibiosis mechanisms of resistance to turnip aphid, *Lipaphis erysimi* (Kaltenbach) in *Brassica juncea-fruticulosa* introgression lines. J. Pestic. Sci. 95, 749–760. doi: 10.1007/s10340-021-01418-8

[B25] Pasiecznik N. M. Smith I. M. Watson G. W. Brunt A. A. Ritchie B. Charles L. M. F. (2005). CABI/EPPO distribution maps of plant pests and plant diseases and their important role in plant quarantine. Bull. Oepp. 35, 1–7. doi: 10.1111/j.1365-2338.2005.00815.x

[B26] Patel S. R. Awasthi A. K. Tomar R. K. S. (2004). Assessment of yield losses in mustard (*Brassica juncea* l.) due to mustard aphid (*Lipaphis erysimi* kalt.) under different thermal environments in Eastern central India. Appl. Ecol. Environ. Res. 2, 1–15. doi: 10.15666/aeer/02001015

[B27] Pearson R. G. Raxworthy C. J. Nakamura M. Townsend Peterson A. (2007). Predicting species distributions from small numbers of occurrence records: a test case using cryptic geckos in Madagascar. J. Biogeogr. 34, 102–117. doi: 10.1111/j.1365-2699.2006.01594.x

[B28] Pekas A. Craecker D. I. Boonen S. Wäckers F. L. Moerkens R. (2020). One stone; two birds: concurrent pest control and pollination services provided by aphidophagous hoverflies. Biol. Control. 149, 104328. doi: 10.1016/j.biocontrol.2020.104328

[B29] Phillips S. J. Anderson R. P. Schapire R. E. (2006). Maximum entropy modeling of species geographic distributions. Ecol. Modell. 190, 231–259. doi: 10.1016/j.ecolmodel.2005.03.026

[B30] Phillips S. J. Dudík M. (2008). Modeling of species distributions with maxent: new extensions and a comprehensive evaluation. Ecography. 31, 161–175. doi: 10.1111/j.0906-7590.2008.5203.x

[B31] Putra N. S. Yasuda H. (2006). Effects of prey species and its density on larval performance of two species of hoverfly larvae, *Episyrphus balteatus* de geer and *Eupeodes corollae* fabricius (Diptera: Syrphidae). Appl. Entomol. Zool. 41, 389–397. doi: 10.1303/aez.2006.389

[B32] Rana J. S. (2005). Performance of *Lipaphis erysimi* (Homoptera: Aphididae) on different brassica species in a tropical environment. J. Pestic. Sci. 78, 155–160. doi: 10.1007/s10340-005-0088-3

[B33] Reza M. D. W. Biswas A. K. Roy K. (2004). Seasonal abundance of *Lipaphis erysimi* (Kalt.) population on mustard. Uttar. Pradesh J. Zoology. 24, 129–132.

[B34] Sillero N. (2011). What does ecological modelling model? a proposed classification of ecological niche models based on their underlying methods. Ecol. Modell. 222, 1343–1346. doi: 10.1016/j.ecolmodel.2011.01.018

[B35] Singh R. Singh D. Rao V. U. M. (2007). Effect of abiotic factors on mustard aphid (*Lipaphis erysimi* kalt.) on Indian brassica. Indian J. Agric. Sci. 41, 67–70.

[B36] Van Vuuren D. P. Edmonds J. Kainuma M. Riahi K. Thomson A. Hibbard K.. (2011). The representative concentration pathways: an overview. Clim. Change. 109, 5–31. doi: 10.1007/s10584-011-0148-z

[B37] Wang R. Yang H. Luo W. Wang M. Lu X. Huang T.. (2019). Predicting the potential distribution of the Asian citrus psyllid, *Diaphorina citri* (Kuwayama), in China using the MaxEnt model. PeerJ. 7, e7323. doi: 10.7717/peerj.7323 31341749 PMC6637924

[B38] Warren D. L. Seifert S. N. (2011). Ecological niche modeling in maxent: the importance of model complexity and the performance of model selection criteria. Ecol. Appl. 21, 335–342. doi: 10.1890/10-1171.1 21563566

[B39] Welch K. D. Harwood J. D. (2014). Temporal dynamics of natural enemy–pest interactions in a changing environment. Biol. Control. 75, 18–27. doi: 10.1016/j.biocontrol.2014.01.004

[B40] Yackulic C. B. Chandler R. Zipkin E. F. Royle J. A. Nichols J. D. (2013). Presence-only modelling using MAXENT: when can we trust the inferences? Methods Ecol. Evol. 4, 236–243. doi: 10.1111/2041-210x.12004

[B41] Yamamura K. Yokozawa M. Nishimori M. Ueda Y. Yokosuka T. (2006). How to analyze long-term insect population dynamics under climate change: 50-year data of three insect pests in paddy fields. Popul. Ecol. 48, 31–48. doi: 10.1007/s10144-005-0239-7

[B42] Zhang Y. Hughes A. C. Zhao Z. Li Z. Qin Y. (2022b). Including climate change to predict the global suitable area of an invasive pest: *Bactrocera correcta* (Diptera: Tephritidae). Glob. Ecol. Conserv. 34, e02021. doi: 10.1016/j.gecco.2022.e02021

[B43] Zhang Q. C. Wang J. G. Lei Y. H. (2022a). Predicting distribution of the Asian longhorned beetle, anoplophora glabripennis (Coleoptera: Cerambycidae) and its natural enemies in China. Insects 13, 687. doi: 10.3390/insects13080687 36005312 PMC9409243

[B44] Zhao Q. Gao X. Y. Cai B. Chen C. Wei J. F. (2022a). Potential distribution of *Diatraea saccharalis* in China based on max ent model. Plant Quarantine 36, 77–82. doi: 10.19662/j.cnki.issn1005-2755.2021.00.032

[B45] Zhao Y. Wang J. G. Chen C. Mao Q. Y. Huang H. C. Cai H.. (2022b). Study on the potential distribution area of *Diaphorina citri* (Kuwayama) in yunnan based on max ent niche model. J. Yunnan Agric. Univ. (Natural Science). 37, 61–68. doi: 10.12101/j.issn.1004-390X(n).202012003

